# Molecular Typing and Epidemiology of Human Listeriosis Cases, Denmark, 2002–2012^1^

**DOI:** 10.3201/eid2204.150998

**Published:** 2016-04

**Authors:** Anne Kvistholm Jensen, Jonas T. Björkman, Steen Ethelberg, Kristoffer Kiil, Michael Kemp, Eva Møller Nielsen

**Affiliations:** Statens Serum Institut, Copenhagen, Denmark (A.K. Jensen, J.T. Björkman, S. Ethelberg, K. Kiil, E.M. Nielsen);; University of Southern Denmark, Odense, Denmark (A.K. Jensen, M. Kemp);; Odense University Hospital, Odense (M. Kemp)

**Keywords:** listeriosis, Listeria monocytogenes, epidemiology, molecular epidemiology, pulsed-field gel electrophoresis, multilocus sequence typing, foodborne diseases, bacteria, humans, Denmark, zoonoses

## Abstract

Denmark has a high incidence of invasive listeriosis (0.9 cases/100,000 population in 2012). We analyzed patient data, clinical outcome, and trends in pulsed-field gel electrophoresis (PFGE) and multilocus sequence typing (MLST) of *Listeria monocytogenes* strains isolated in Denmark during 2002–2012. We performed 2-enzyme PFGE and serotyping on 559 isolates and MLST on 92 isolates and identified some correlation between molecular type and clinical outcome and patient characteristics. We found 178 different PFGE types, but isolates from 122 cases belonged to just 2 closely related PFGE types, clonal complex 8 and sequence type 8. These 2 types were the main cause of a peak in incidence of invasive listeriosis during 2005–2009, possibly representing an outbreak or the presence of a highly prevalent clone. However, current typing methods could not fully confirm these possibilities, highlighting the need for more refined discriminatory typing methods to identify outbreaks within frequently occurring *L. monocytogenes* PFGE types.

## Abstract 2

A clone of *Listeria monocytogenes* CC8 caused bacteremia in the elderly and a high incidence of listeriosis.

Listeriosis is a foodborne infection that can cause life-threatening illnesses, including bloodstream infections (BSI) and central nervous system (CNS) infections. Listeriosis is caused by the Gram-positive and ubiquitous bacterium *Listeria monocytogenes* and mainly affects the elderly, immunocompromised persons, and pregnant women. Even though pregnant women often have mild or no clinical symptoms*, L. monocytogenes* can cause severe systemic infection in fetuses and neonates; infection in utero can lead to preterm birth or abortion and stillbirth (*1*).

Most listeriosis cases are sporadic, but outbreaks do occur. Ready-to-eat products, such as delicatessen meats, soft cheeses and smoked seafood, have repeatedly been identified by foodborne disease control programs as sources of outbreaks and products that put humans at risk for listeriosis (*2*–*4*). For surveillance of outbreaks and trace-back of contaminated sources, highly discriminative molecular subtyping methods are instrumental in supporting the epidemiologic investigation. Pulsed-field gel electrophoresis (PFGE) has been considered the standard typing method for *L. monocytogenes* (*5*,*6*). The advantage of PFGE is mainly its high discriminative power. However, compared with sequence-based typing methods, such as multilocus sequence typing (MLST), PFGE has ambiguity in interpreting data and lacks standard nomenclature; thus, PFGE has difficulties in readily comparing international data. In addition, MLST provides information on phylogenetic relationship. *L. monocytogenes* is genetically heterogeneous and can be divided into 4 genetic lineages (I–IV) that have different pathogenic properties. Isolates from food and from human cases most frequently belong to lineages I and II (*7*).

In Denmark, the annual incidence of listeriosis increased from 0.5 cases per 100,000 population in 2002–2003 to a peak of 1.8 cases in 2009 and 0.9 cases in 2012, and is now among the highest incidences reported globally (*8*,*9*). Similar increasing trends have been reported from other European countries during the same period (*4*). The high but variable incidence calls for further examination of the possible explanations. We retrospectively analyzed trends related to patient data and PFGE- and MLST-types of *L. monocytogenes* strains occurring in Denmark during 2002–2012. In addition, we assessed the possible association between clinical aspects of the disease and strain genotype.

## Materials and Methods

### Case Information

This study comprises all culture-confirmed cases of invasive listeriosis in humans in Denmark during 2002–2012. In Denmark, listeriosis is notifiable by diagnostic laboratories to the reference laboratory at Statens Serum Institut (SSI) in Copenhagen. Reportable information is patient age and sex, sample isolation site, date of specimen collection, and hospital and hospital department at which the specimen was collected. The case definition for listeriosis used in Denmark is in accordance with the case definition by the European Commission (*10*). Cases are categorized according to the site of isolation of *L. monocytogenes* (usually blood or cerebrospinal fluid), clinical diagnosis, or both. Hence, case-patients are grouped according to those with BSIs, CNS infections, pregnancy-associated infections, or other infections. Pregnancy-associated infections comprise listeriosis in neonates in the first month of life and maternal–fetal infections. A pregnancy-associated infection is counted as a single case and is reported as an infection in the mother. From the Danish Civil Registry System, we collected information on vital status to estimate the case-fatality rate (CFR) for non–pregnancy-associated cases. We defined a fatal case as death occurring within 30 days of the date the diagnostic specimen was collected.

### Characterization of Bacterial Isolates

The diagnostic laboratories not only notify SSI of listeriosis cases but also refer isolated bacteria to SSI for phenotypic confirmation and typing. Serotypes were established by agglutination with BD Difco Listeria O Antisera Type 1 and 4 (Becton, Dickinson and Company, Sparks, MD, USA) on all isolates and with PCR serogrouping on a subset of isolates (*11*). During 2006–2012, *L. monocytogenes* isolates from human cases were routinely typed by PFGE at the SSI as a means to survey for outbreaks. PFGE was performed according to the PulseNet protocol, using the restriction enzymes *Asc*I and *Apa*I (*6*). For the isolates from 2002 through 2005, PFGE was performed retrospectively as part of this study. We used BioNumerics software version 6.6 (Applied Maths, Sint-Martens-Latum, Belgium) to analyze gels and assign bands. The combined *Asc*I and *Apa*I profiles defined the pulsotype. Isolates were considered having the same pulsotype if they had identical band patterns (no single-band differences) with both enzymes. We calculated similarity between band patterns by using the Dice coefficient, with optimization and tolerance set at 1% for both enzymes. We constructed a dendrogram based on the combined *Asc*I and *Apa*I profiles using UPGMA. Based on this dendrogram, MLST, and PCR serogroup, the genetic lineage of each isolate was determined (*7*,*12*).

We selected 92 isolates for MLST analysis. Isolates were selected in proportion to the number of cases from each year, the different clinical manifestations, the distribution of geography and age groups, and so that they represented both common and rare pulsotypes (Technical Appendix Figure 1). For 74 isolates, MLST alleles were extracted from whole-genome sequencing data from Illumina platforms (Illumina Inc., San Diego, CA, USA) by mapping the raw reads to each of the 7 MLST loci sequences from *L. monocytogenes* strain EDG-e. Mapping was done using an in-house pipeline based on BWA (Burrows–Wheeler Aligner, http://bio-bwa.sourceforge.net/) and SAMtools (Sequence Alignment/Map, http://www.htslib.org/). For 18 isolates, MLST alleles were determined by conventional PCR and Sanger sequencing according to the MLST scheme by Ragon et al. (*12*). MLST sequence type (ST) and clonal complex (CC) were assigned by using the Institut Pasteur *L. monocytogenes* MLST sequence type database (http://www.pasteur.fr/recherche/genopole/PF8/mlst/Lmono.html). Isolates of the same 2-enzyme pulsotype generally belong to the same CC and, in most instances, the same ST (*13*); therefore, isolates of the same pulsotypes as those typed by MLST were assigned to a presumptive ST and CC. We defined a cluster as the occurrence of at least 3 cases with indistinguishable pulsotypes within a period of 14 weeks (*14*).

### Statistical Analyses

Difference in age between groups of patients was assessed by using the Wilcoxon rank-sum test. Categorical variables were compared using χ^2^ or Fisher exact test, when appropriate. Relative risks (RRs) with accompanying 95% CIs were calculated; p<0.05 using 2-sided tests indicated statistical significance. We used SAS version 9.4 software (SAS Institute, Inc., Cary, NC, USA) for statistical calculations.

## Results

### Description of Cases and Origin of Bacterial Isolates

In Denmark, 570 cases of invasive listeriosis were notified during 2002–2012; of these, 52% were in women. All patients were hospitalized. For 559 (98%) cases, an isolate of *L. monocytogenes* was referred to SSI. Of the infections, 73% were BSIs, 19% were CNS infections, 4% were pregnancy-associated infections, and 4% were other infections (Table 1). On average, the proportion of BSIs varied from 69% in 2002–2004 to 78% in 2005–2009 and fell to 66% in 2010–2012; the proportion of CNS infections increased from 16% in 2002–2004 to 25% in 2010–2012. Median age of patients with non–pregnancy-associated infections was 71 years; no difference in age was found between patients with BSIs and CNS infections. Of all registered cases, 150 resulted in death within 30 days of the sample collection date; 95 of patients with fatal disease were >70 years of age. The overall CFR for non–pregnancy-associated cases was 27% (range 17%–40%, by year). CFR varied with age: 22% for patients <70 years of age versus 33% for patients >70 years (p = 0.004). Overall, we observed similar CFRs for CNS infections and BSIs and for male and female patients.

**Table 1:** Characteristics of reported cases of human listeriosis by year, Denmark, 2002–2012*

Year	No. cases reported	Incidence per 10^5^ population	Median patient age, y (range)†	Infection type, no. (%)				CFR, %†
				CNS	BSI	Pregnancy-associated	Other‡	
2002	28	0.52	69 (1–90)	6 (21)	18 (64)	1 (4)	3 (11)	26
2003	28	0.52	76 (23–95)	5 (18)	18 (64)	3 (11)	2 (7)	24
2004	41	0.76	74 (44–98)	5 (12)	31 (76)	4 (10)	1 (2)	27
2005	44	0.81	68 (23–95)	4 (9)	39 (89)	0 (0)	1 (2)	23
2006	58	1.07	70 (8–91)	13 (22)	43 (74)	2 (2)	0 (0)	20
2007	59	1.08	67 (19–96)	11 (19)	45 (76)	0 (0)	3 (5)	32
2008	55	1.00	70 (1–93)	4 (7)	45 (82)	1 (2)	5 (9)	33
2009	98	1.78	74 (44–98)	18 (18)	74 (76)	4 (4)	2 (2)	29
2010	61	1.10	75 (2–91)	15 (25)	40 (66)	6 (10)	0 (0)	40
2011	48	0.86	70 (2–96)	9 (19)	36 (75)	0 (0)	3 (6)	25
2012	50	0.90	74 (24–93)	15 (30)	30 (60)	2 (4)	3 (6)	17
Total	570	0.95	71 (1–98)	105 (18)	419 (74)	23 (4)	23 (4)	27

*BSI, blood stream infection; CFR, case-fatality rate; CNS, central nervous system. †Calculated for non–pregnancy-associated cases only. ‡Includes peritonitis, pleuritis, arthritis, abscesses, and osteitis.

### Genetic Lineage and Serotype

Cluster analysis of combined *Asc*I and *Apa*I pattern divided the isolates into 3 genetic lineages (Technical Appendix Figure 1), as did PCR serogrouping and MLST. We found that 42% of isolates belonged to lineage I and 58% to lineage II; 1 isolate belonged to lineage III/IV (PCR serogroup L). Within the lineage I isolates, 82% (193/235) were serotype 4 and 18% (42/235) were serotype 1. Of the 42 serotype 1 lineage I isolates, 12 were typed by PCR serogrouping and were PCR serogroup IIb. All lineage II isolates belonged to serotype 1, predominantly serogroup 1/2a, as determined by PCR serogroup (Technical Appendix Figure 1).

### Molecular Typing

**Figure 1:**
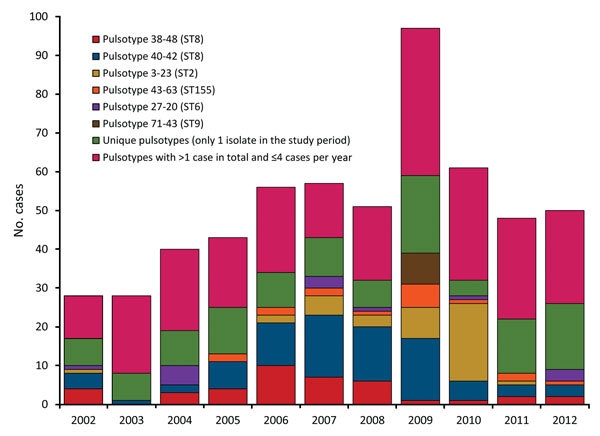
Distribution of pulsotypes of *Listeria monocytogenes* isolates from humans with listeriosis, Denmark, 2002–2012.

PFGE divided the strains into 122 *Asc*I and 140 *Apa*I profiles, for a total of 178 combinations (pulsotypes), which we identified by year (Figure 1). During the 11-year period, 116 pulsotypes (representing 21% of typed cases) occurred only once, and 56 pulsotypes (representing 43% of cases) were seen 2–13 times. The 5 most common pulsotypes represented 82, 40, 40, 17, and 14 cases. 

The 92 isolates selected for MLST belonged to 53 pulsotypes and 28 different STs. These 53 pulsotypes represented 69% (387/559) of all reported isolates. The 10 most frequent STs among these 387 isolates were CC8/ST8 (121 cases), CC2/ST2 (52 cases), CC6/ST6 (48 cases), CC1/ST1 (36 cases), CC9/ST9 (22 cases), CC155/ST155 (27 cases), CC7/ST7 (15 cases), and CC5/ST5 and CC14/ST399 (10 cases each) (Technical Appendix Figure 2). 

**Figure 2:**
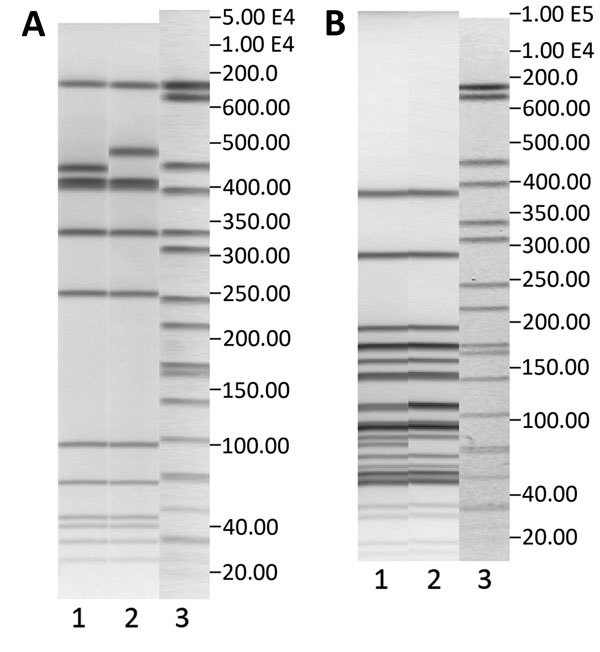
Pulsed-field gel electrophoresis (PFGE) profiles with *Asc*I (A) and *Apa*I (B) restriction enzymes of the 2 most common pulsotypes, 40–42 and 38–48, in Denmark, 2002–2012. A) Lane 1, pulsotype 38–48, GX6A16.0038 DK; lane 2, pulsotype 40–42, GX6A16.0040; lane 3, markers. B) Lane 1, pulsotype 38–48, GX6A12.0048 DK; lane 2, pulsotype 40–42, GX6A12.0042 DK; lane 3, markers. Both pulsotypes belong to clonal complex 8.

The 2 most common pulsotypes, 40–42 and 38–48, were represented by 122 (82 and 40, respectively) isolates. These pulsotypes differed by only 2 bands in the *Asc*I pattern and 1 band in the *Apa*I pattern (Figure 2). During 2005–2009, a total of 36% (range 18%–40%) of the isolates belonged to these 2 pulsotypes (Figure 1). Isolates of pulsotypes 40–42 and 38–48 were ST8, except for 1 isolate belonging to ST120. Both ST8 and ST120 belong to CC8. Pulsotype 3–23, which was found in 40 cases and mostly seen in 2009–2010, was CC2/ST2.

### Relationship between Typing and Epidemiologic Data

We summarized the subtyping and clinical data related to the pulsotypes associated with >8 cases in this study (Table 2). Isolates of pulsotypes belonging to genetic lineage I generally were responsible for a higher proportion of CNS and pregnancy-associated cases than isolates of pulsotypes belonging to genetic lineage II. Isolates belonging to the 2 most common pulsotypes, 40–42 and 38–48, which belong to CC8, were responsible for high proportions of BSIs, corresponding to the high number of BSIs seen in the years when these types were predominant. Of note, none of the CC8 isolates caused pregnancy-associated cases. In contrast, isolates of pulsotype 3–23 (CC2/ST2) alone caused 6 pregnancy-associated cases in 2009–2010. The remaining 17 pregnancy-associated cases were caused by 14 pulsotypes.

**Table 2:** Clinical and subtype data associated with the most common PFGE pulsotypes of *Listeria monocytogenes* isolates from persons with listeriosis, Denmark, 2002–2012*

Pulsotype	ST/CC	Genetic lineage (PCR serogroup)	No. cases	Median patient age, y (range)†	Infection type, no. (%)				CFR, %†
					BSI	CNS	Pregnancy-associated	Other‡	
40–42	8/8	II (IIa)	82	71 (8–91)	68 (83)	11 (13)	0 (0)	3 (4)	30
38–48	8/8§	II (IIa)	40	69 (24–91)	31 (78)	7 (18)	0 (0)	2 (5)	25
3–23	2/2	I (IVb)	40	69 (1–90)	23 (58)	11 (28)	6 (15)	0 (0)	29
43–63	155/155	II (IIa)	17	75 (42–95)	15 (88)	1 (6)	0 (0)	1 (6)	41
27–20	6/6	I (IVb)	14	74 (57–98)	11 (79)	3 (21)	0 (0)	0 (0)	29
25–1	1/1	I (IVb)	13	67 (2–83)	6 (46)	6 (46)	0 (0)	1 (8)	23
41–43	9/9	II (IIc)	11	72 (53–93)	9 (82)	1 (9)	0 (0)	1 (9)	45
22–2	1/1	I (IVb)	11	72 (37–90)	5 (46)	4 (36)	2 (18)	0 (0)	11
44–61	155/155	II (IIa)	10	76 (49–91)	7 (70)	2 (20)	1 (10)	0 (0)	33
71–43¶	9/9	II (IIa)	8	77 (44–94)	7 (88)	1 (13)	0 (0)	0 (0)	25
67–92	1/1	I (IVb)	8	74 (63–98)	7 (88)	1 (13)	0 (0)	0 (0)	25
27–18	6/6	I (IVb)	8	52 (50–76)	3 (38)	4 (50)	1 (13)	0 (0)	29
12–38	7/7	II (IIa)	8	73 (51–90)	3 (38)	5 (63)	0 (0)	0 (0)	13
35–26	59/59	I (IIb)	8	70 (55–80)	4 (50)	2 (25)	2 (25)	0 (0)	17

*Data are for pulsotypes found in >8 cases. Pulsotype is for combined *Asc*I and *Apa*I pattern. BSI, blood stream infection; CC, clonal complex; CFR, case-fatality rate; CNS, central nervous system; PFGE, pulsed-field gel electrophoresis; ST, sequence type. †Calculated for non–pregnancy-associated cases only. ‡Includes peritonitis, pleuritis, arthritis, abscesses, and osteitis. §One of the 5 representative isolates selected for multilocus sequence typing for this pulsotype was ST120/CC8. ¶Confirmed outbreak described previously (*15*).

**Figure 3:**
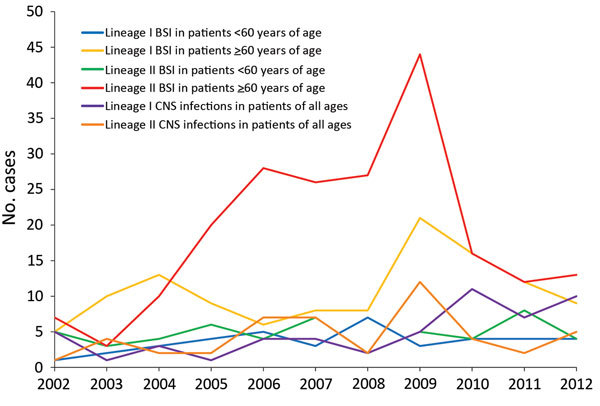
Distribution of lineages of *Listeria monocytogenes* isolates by disease manifestation, Denmark, 2002–2012. Only blood stream infections (BSIs) and central nervous system (CNS) infections are shown. For BSIs, the distribution by age is also shown.

Over time, the distribution of lineages by clinical manifestation (BSI and CNS infection) and age (for BSI) revealed that the number of BSIs in patients >60 years of age, especially with lineage II isolates, showed an increasing tendency, peaking in 2009 and then declining (Figure 3). Overall, a slightly increasing tendency was seen for CNS infections caused by lineage I isolates.

We found a statistically significant difference in the clinical manifestation of disease caused by lineage I and II. BSIs were more common in patients infected with lineage II strains than in those infected with lineage I strains (79.8% vs. 66.8%, respectively; p<0.001), CNS infections were more common among patients with lineage I strains than those with lineage II strains (22.5% vs. 14.9%, respectively; p<0.001), and more pregnancy-associated cases were caused by lineage 1 than lineage II strains (7.7% vs. 1.6%, respectively; p<0.001). We found no difference in the age of patients infected with isolates of genetic lineage I or II; median age was 71 years for both groups of patients. We observed a moderate effect of genetic lineage on the CFR; 29.6% of patients infected with lineage II strains died, versus 23.0% infected with lineage I strains.

The RR of death from infection with a lineage II strain compared with a lineage I strain was 1.28 (95% CI 0.95–1.73). However, among persons with CNS infections, the effect of lineage was greater (RR 3.31; 95% CI 1.55–7.09), but age was an effect modifier in the sense that for patients <70 years of age, the RR of death from CNS infection with a lineage II strain, versus a lineage I strain, was 6.07 (95% CI 1.44–25.45), and for patients >70 years of age, the RR was 2.11 (95% CI 0.88–5.05). Effects of lineage and age on CFR were not seen among patients with BSIs.

### Cluster Detection

In the retrospective analysis of PFGE data, we detected 29 clusters comprising 174 cases (Table 3). The 3 most frequent pulsotypes each formed more than 1 cluster. Overall, 13 clusters involved the 2 pulsotypes belonging to CC8, and 8 of the 13 clusters occurred during 2005–2009 where these 2 types were highly prevalent. We found 4 clusters defined by pulsotypes that were seen only at the time of the cluster (n = 3) or in the year the cluster was present (n = 1). One of these 4 clusters, the cluster with pulsotype 71–43, was a confirmed outbreak (*15*).

**Table 3:** Clusters of identical pulsotypes of *Listeria monocytogenes* isolates, Denmark, 2002–2012*

Year(s)	No. cases	Time range for the cluster, d	Genetic lineage (PCR serogroup)	ST/CC	Pulsotype
Clusters with pulsotypes other than the 3 highly frequent types					
2003†	3	40	I (ND)	224/224	181–218
2004	3	90	I (IVb)	6/6	27–20
2005‡	3	97	I (ND)	5/5	31–30
2005–2006	3	42	I (IVb)	1/1	25–1
2006	3	66	II (IIa)	155/155	44–61
2006†	4	103	II (ND)	391/89	21–34
2009	4	41	II (IIa)	155/155	43–63
2009†§	8	5	II (IIa)	9/9	71–43
2009	4	130	I (IIb)	59/59	35–26
2009	3	97	I (IVb)	1/1	67–92
2010	3	60	II (IIc)	9/9	41–43
2010	3	28	I (IVb)	1/1	22–2
2011	4	108	II (IIa)	18/18	101–126
Clusters with the 3 highly frequent pulsotypes					
2004	3	84	II (IIa)	8/8	38–48
2005	3	81	II (IIa)	8/8	38–48
2006–2007	11	191	II (IIa)	8/8	38–48
2007	6	164	II (IIa)	8/8	38–48
2008	3	81	II (IIa)	8/8	38–48
2005	5	86	II (IIa)	8/8	40–42
2006–2007	12	230	II (IIa)	8/8	40–42
2007–2008	23	421	II (IIa)	8/8	40–42
2008–2009	6	83	II (IIa)	8/8	40–42
2009–2010	15	310	II (IIa)	8/8	40–42
2010	3	44	II (IIa)	8/8	40–42
2011	3	55	II (IIa)	8/8	40–42
2012	3	75	II (IIa)	8/8	40–42
2007	3	46	I (IVb)	2/2	3–23
2009–2010¶	8	148	I (IVb)	2/2	3–23
2010#	19	253	I (IVb)	2/2	3–23

*A cluster was defined as >3 cases with identical pulsotypes within 14 weeks. ST, sequence type; CC, clonal complex; ND not determined. †This type only seen this 1 time during the study period. ‡This type only seen in 2005. §Confirmed outbreak with meals-on-wheels food delivery as source of infection, described previously (*15*). ¶Cluster investigated but no common source identified. #May be part of the former cluster (marked with ¶) with this pulsotype 3–23.The cluster included 5 pregnancy-associated cases. Cluster investigation could not determine a common source of infection, but smoked salmon was suspected based upon interview data.

## Discussion

This study provides insights into the dynamics of the pulsotypes of *L. monocytogenes* isolates through a period with an increasing incidence of listeriosis in Denmark. We found that during 2005–2009, an increase in the number of cases was mainly driven by the emergence of isolates of *L. monocytogenes* of genetic lineage II (serotype 1/2a). Throughout the study period, we found a high proportion of sporadic cases caused by unique or infrequent pulsotypes. Nevertheless, a remarkably high proportion, 36%, of the infections in 2005–2009 were caused by isolates of CC8 with 2 closely related pulsotypes.

CC8 is globally distributed (*16*). In Switzerland, CC8 was the most prevalent clone during 2011–2013 (*17*), and in Canada, a CC8/ST120 clone caused both sporadic cases and outbreaks during 1988–2010 (*18*). The PFGE types of the CC8 isolates from Canada are quite similar to the CC8 pulsotypes reported in our study (*19*). In line with the epidemiologic findings in Canada, the CC8 clone in Denmark caused no pregnancy-associated infections and mostly caused infections in the elderly. This propensity for CC8 pulsotypes to infect older persons rather than pregnant women may reflect different food preferences between the 2 groups at risk for listeriosis as well as the frequency of exposure to food substances contaminated with CC8 *L. monocytogenes*; alternatively, this propensity may be related to the virulence potential of this specific clone. Recent research has suggested that the CC8 strains from Canada possess a strong capacity for biofilm formation, which may support persistence within food production environments and subsequent contamination of foods (*20*). Similar abilities could be harbored by the CC8 isolates from Denmark, but further examination is needed to confirm this. 

Nakari et al. (*21*) found that an increase in listeriosis in Finland in 2010 was partly caused by a specific *Asc*I type, *Lm*96, which caused 19% of human cases and was the most prevalent type found in food isolates in Finland in 2010. Of note, *Lm*96 was identical to the *Asc*I profile 38 in Denmark, the second most common *Asc*I profile belonging to CC8. The type was found in a specific fishery production plant in Finland and in cold-smoked rainbow trout products from the plant. A persistent contamination of the plant was suspected because the same PFGE type had been found in earlier years. An enquiry via the Food and Waterborne Diseases Network revealed that *Lm*96 was a common profile in several European countries and had been isolated from various food categories in addition to fishery products (*21*).

The fact that closely related or even identical pulsotypes have been found in the same period raises the question whether the contamination of food could originate from a common source (e.g., a fish farm). Studies have shown that *L. monocytogenes* isolates found in fish farms and raw fish material often belonged to the same pulsotypes as isolates from the final product, indicating that raw fish material can be important sources of *L. monocytogenes* contamination of final fishery products (*22*,*23*). For several years, cold-smoked fishery products have been among the important suspected sources of infections in Denmark. Ready-to-eat fishery products are consumed in large amounts and are the food category most often found to be contaminated with *L. monocytogenes* in the European Union (*3*,*4*,*24*). Lambertz et al. (*25*) compared isolates of *L. monocytogenes* recovered in 2010 from ready-to-eat foods and processing plants in Sweden with clinical isolates obtained from listeriosis patients during 2005–2010. They found that the most common human pulsotype was also the most common pulsotype among the isolates from ready-to-eat foods, and 17 of 19 food isolates of this pulsotype originated from fishery products from a processing plant not in Sweden. Similar findings were made in Norway by Lunestad et al. (*26*). Whether the CC8 isolates described in our study reflect the presence of a common pulsotype with many unrelated sources or a long-term cluster with a common source of infection is difficult to determine.

Since 2006 in Denmark, 2-enzyme PFGE has been used to type human isolates of *L. monocytogenes*. Listeriosis clusters caused by *L. monocytogenes* pulsotypes 40–42 and 38–48 were defined separately and restricted to predefined periods of weeks. During 2006–2007, basic epidemiologic information was collected and, if possible, patients were contacted, but no standardized questionnaire existed. However, no correlation between cases and a common source of infection was established, and it was concluded the cases were caused by common *L. monocytogenes* pulsotypes (Steen Ethelberg, unpub. data). Our findings show that the prevalence of both pulsotypes increased during 2005–2009 and substantially decreased beginning in 2010. This pattern could indicate that a common source caused infections through several years. Some pulsotypes are more common than others, and isolates of these pulsotypes might not be epidemiologically linked. Consequently, common pulsotypes create challenges in defining cluster detection levels (*27*). Our findings show that with a cluster detection level of 3 cases with identical pulsotypes within a period of 14 weeks, possible true clusters of rare pulsotypes are discovered, but the number of detected clusters associated with common pulsotypes is apparently overestimated. Having different cluster detection levels based on the frequency of the pulsotypes may enhance the chance of detecting true clusters (*27*,*28*). On the other hand, because clones of *L. monocytogenes* can persist in food processing plants for several years, exhibiting little genetic variation and causing infections spanning several years (*29*), narrow temporal cluster definitions may not always be appropriate for cases of listeriosis with identical pulsotypes. Our results point to the need for typing methods that can provide higher resolution of common pulsotypes but also phylogenetically link related pulsotypes and, thus, improve the epidemiologic investigations of suspected outbreaks. Whole-genome sequencing seems to be able to fulfill these needs, as has already been shown in several outbreaks with listeriosis (*19*,*30*,*31*). In addition, optimizing epidemiologic information through routine interviews of all cases of listeriosis has also proven powerful in finding common sources of infections as well as defining outbreaks involving strains of different genotypes (*2*,*32*,*33*). Last, real-time comparison of subtyping results between isolates recovered from foods and humans would substantially increase the knowledge on possible sources of infection and enhance the chances of successful outbreak investigations.

We found a substantial increase in BSIs among patients >60 years of age with lineage II isolates during 2005–2009, which, for the most part, explained the increased incidence seen in those years. Our findings concur with those from studies in England and Wales, in which Gillespie et al. (*34*) found that an increase in cases of listeriosis in 2001–2007 was mainly related to persons >60 years of age with bacteremia. Similarly, other European countries have reported an increase in infections caused by serotype 1/2a (*35*,*36*). Our study did not include data on concurrent conditions, socioeconomic factors, or medications used by persons with listeriosis, which could have contributed with further explanatory variables, as in the study by Gillespie et al. (*34*), in which patients with cancer and patients receiving stomach acid inhibitors were mainly affected by the increase.

We found some associations between specific molecular types and clinical manifestations. The pulsotype 3–23, belonging to CC2/ST2 and serotype 4b, accounted for 6 of 23 pregnancy-associated infections; 5 of these occurred in autumn 2010 and were probably linked. Pulsotypes belonging to CC1, CC2, and CC6 showed high proportions of CNS infections, which is in accord with our finding that lineage I was associated with more CNS infections and pregnancy-associated cases. In contrast with a previous study from Denmark (*37*), we found that the CFR for patients infected with lineage II (serotype 1/2a and 1/2c) isolates was slightly higher than that for patients infected with lineage I (serotype 4b and 1/2b) isolates. Compared with lineage I, lineage II was significantly associated with a higher mortality rate for patients <70 years of age with CNS infections but not for patients >70 years of age with CNS infections. However, this study would have been strengthened if we had had information on concurrent conditions, as they could be confounding this association.

Our findings show that retrospective typing of isolates gives new insight into the epidemiology of listeriosis. By PFGE typing, we found a high diversity of *L. monocytogenes* in clinical cases but also a small number of frequent types representing a substantial fraction of all cases. Possibly, these types represent epidemiologically linked cases (outbreaks) or, alternatively, ubiquitous types present in many unrelated food sources and infections. New discriminatory typing methods are necessary to clarify the clonality of these common types. In the near future, whole-genome sequencing is likely to be the method of choice for such analyses. Several studies have reported on the genetic diversity of *L. monocytogenes* based on MLST, making it possible to compare typing data globally (*13*,*16*,*36*,*38*). By the addition of MLST, we could compare types our study with those in other countries, making it clear that some of the common clones in our study had also been found in other countries, thus paving the way for a better understanding of internationally occurring clones. To enhance the surveillance of listeriosis, continuous typing with highly discriminatory methods combined with timely collection of patients’ histories of food intake could significantly improve the chances of detecting, solving, and stopping outbreaks. Moreover, human and food isolates should be typed by the same methods and compared on a regular basis.

Technical Appendix Dendrograms of pulsed-field gel electrophoresis profiles for *Listeria monocytogenes* strains isolated from humans, Denmark, 2002–2012.
