# Supplementary material for: Molecular Typing and Epidemiology of Human Listeriosis Cases, Denmark, 2002–2012
Source: Emerg Infect Dis. 2016 Apr;22(4):625–33. doi: 10.3201/eid2204.150998 (PMC4806936; doi:10.3201/eid2204.150998)
Supplement: Technical Appendix — Dendrograms of pulsed-field gel electrophoresis profiles for Listeria monocytogenes strains isolated from humans, Denmark, 2002–2012. [file 15-0998-Techapp-s1.pdf]

Article DOI: http://dx.doi.org/10.3201/eid2204.150998

# Molecular Typing and Epidemiology of Human Listeriosis Cases, Denmark, 2002–2012

## Technical Appendix

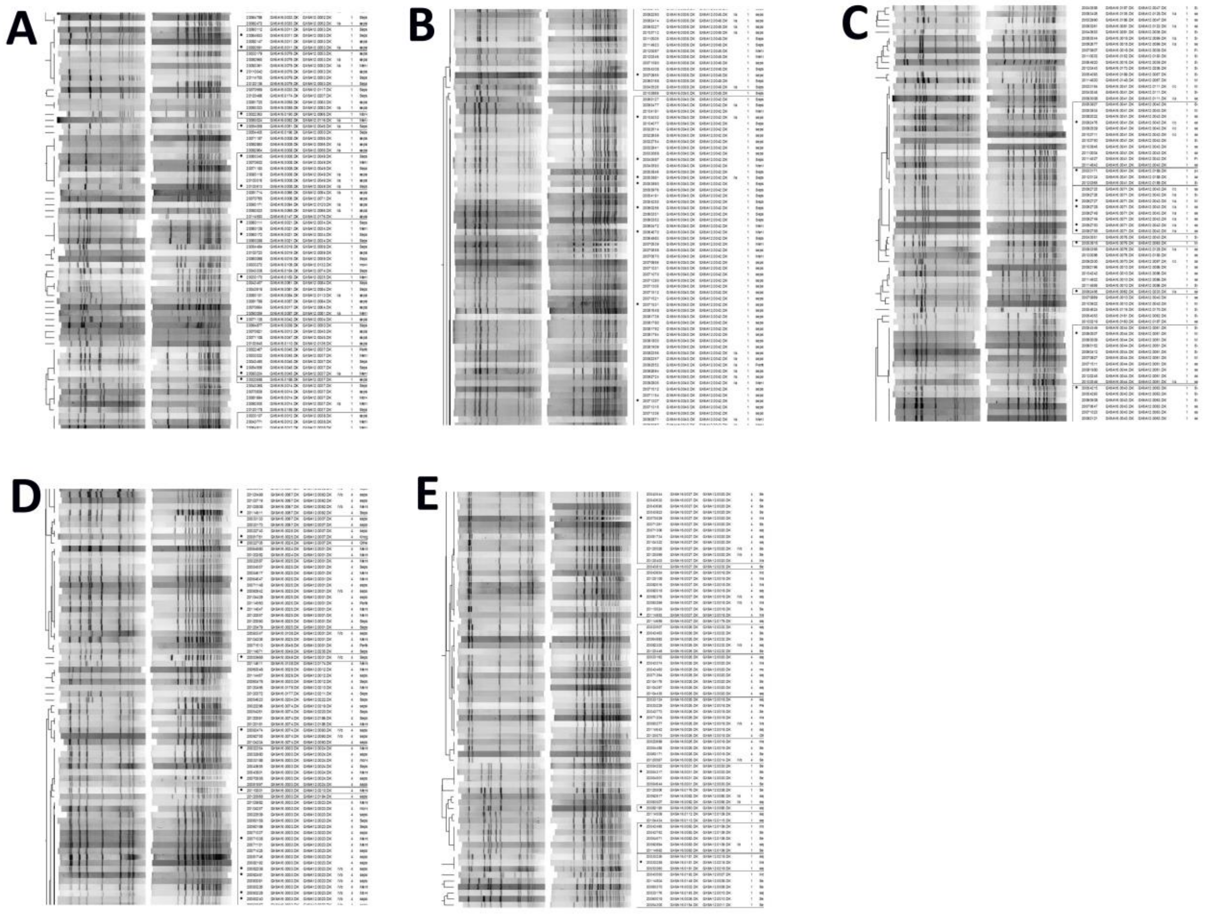


**Technical** Appendix Figure 1. Dendogram of PFGE profiles from the 559 typed isolates in this study. Isolates that have been selected for multilocus sequence typing are marked with" •" and boxes identify isolates of identical pulsotypes to the isolates that have been MLST typed. The clonal complex (CC) and sequence type (ST) are marked for each box. The dotted lines mark separation of lineages. Key, isolate ID, first 4 digits represent the year; Mol Sero, PCR-serogroup; Sero, serotype; Clinical, clinical disease of the case.

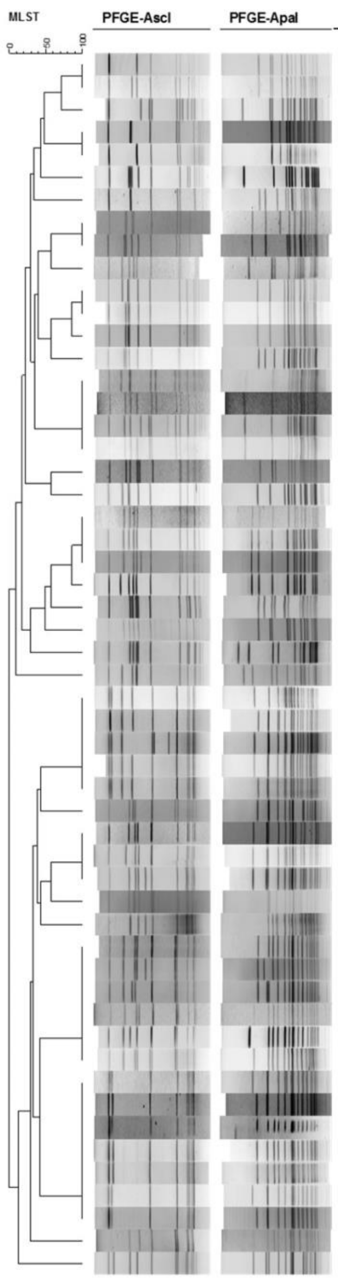


| ST | PFGE-AscI | PFGE-ApaI | Mol Sero | Sero | No. MLST | Total no. pulsotype |
|---|---|---|---|---|---|---|
| 399 | GX6A16.0079.DK | GX6A12.0053.DK | | 1 | 1 | 6 |
| 399 | GX6A16.0011.DK | GX6A12.0053.DK | IIa | 1 | 2 | 4 |
| 400 | GX6A16.0190.DK | GX6A12.0066.DK | | 1 | 1 | 1 |
| 121 | GX6A16.0008.DK | GX6A12.0049.DK | IIa | 1 | 2 | 6 |
| 121 | GX6A16.0051.DK | GX6A12.0046.DK | IIa | 1 | 1 | 1 |
| 18 | GX6A16.0101.DK | GX6A12.0126.DK | | 1 | 1 | 6 |
| 101 | GX6A16.0073.DK | GX6A12.0100.DK | IIa | 1 | 1 | 1 |
| 155 | GX6A16.0043.DK | GX6A12.0063.DK | IIa | 1 | 3 | 17 |
| 155 | GX6A16.0044.DK | GX6A12.0061.DK | | 1 | 1 | 10 |
| 391 | GX6A16.0021.DK | GX6A12.0034.DK | | 1 | 2 | 4 |
| 8 | GX6A16.0040.DK | GX6A12.0042.DK | IIa | 1 | 12 | 82 |
| 8 | GX6A16.0038.DK | GX6A12.0048.DK | | 1 | 4 | 40 |
| 120 | GX6A16.0038.DK | GX6A12.0048.DK | IIa | 1 | 1 | |
| 17 | GX6A16.0183.DK | GX6A12.0225.DK | | 1 | 1 | 1 |
| 7 | GX6A16.0012.DK | GX6A12.0038.DK | IIa | 1 | 2 | 8 |
| 7 | GX6A16.0055.DK | GX6A12.0082.DK | IIa | 1 | 1 | 1 |
| 7 | GX6A16.0188.DK | GX6A12.0037.DK | | 1 | 1 | 1 |
| 7 | GX6A16.0045.DK | GX6A12.0037.DK | IIa | 1 | 2 | 5 |
| 398 | GX6A16.0042.DK | GX6A12.0004.DK | | 1 | 1 | 1 |
| 20 | GX6A16.0040.DK | GX6A12.0237.DK | | 1 | 1 | 2 |
| 9 | GX6A16.0041.DK | GX6A12.0043.DK | IIc | 1 | 1 | 11 |
| 9 | GX6A16.0041.DK | GX6A12.0189.DK | | 1 | 1 | 3 |
| 9 | GX6A16.0071.DK | GX6A12.0043.DK | IIa | 1 | 4 | 8 |
| 713 | GX6A16.0076.DK | GX6A12.0065.DK | | 1 | 1 | 1 |
| 451 | GX6A16.0046.DK | GX6A12.0075.DK | IIa | 1 | 1 | 3 |
| 204 | GX6A16.0041.DK | GX6A12.0135.DK | IIa | 1 | 1 | 1 |
| 124 | GX6A16.0046.DK | GX6A12.0223.DK | | 1 | 1 | 1 |
| 37 | GX6A16.0062.DK | GX6A12.0233.DK | IIa | 1 | 1 | 1 |
| 2 | GX6A16.0003.DK | GX6A12.0210.DK | | 4 | 1 | 1 |
| 2 | GX6A16.0074.DK | GX6A12.0090.DK | IVb | 4 | 1 | 3 |
| 2 | GX6A16.0065.DK | GX6A12.0024.DK | IVb | 4 | 1 | 1 |
| 2 | GX6A16.0003.DK | GX6A12.0024.DK | | 4 | 2 | 7 |
| 2 | GX6A16.0003.DK | GX6A12.0023.DK | IVb | 4 | 8 | 40 |
| 3 | GX6A16.0072.DK | GX6A12.0098.DK | IIb | 1 | 1 | 1 |
| 5 | GX6A16.0060.DK | GX6A12.0085.DK | | 1 | 1 | 1 |
| 5 | GX6A16.0031.DK | GX6A12.0030.DK | | 1 | 1 | 4 |
| 5 | GX6A16.0083.DK | GX6A12.0109.DK | | 1 | 1 | 5 |
| 59 | GX6A16.0035.DK | GX6A12.0026.DK | | 1 | 1 | 8 |
| 426 | GX6A16.0069.DK | GX6A12.0095.DK | IIb | 1 | 1 | 2 |
| 1 | GX6A16.0025.DK | GX6A12.0001.DK | IVb | 4 | 3 | 13 |
| 1 | GX6A16.0067.DK | GX6A12.0092.DK | IVb | 4 | 3 | 8 |
| 1 | GX6A16.0022.DK | GX6A12.0002.DK | IVb | 4 | 1 | 11 |
| 1 | GX6A16.0025.DK | GX6A12.0007.DK | | 4 | 1 | 2 |
| 1 | GX6A16.0049.DK | GX6A12.0031.DK | IVb | 4 | 1 | 1 |
| 1 | GX6A16.0024.DK | GX6A12.0007.DK | | 4 | 1 | 1 |
| 6 | GX6A16.0027.DK | GX6A12.0103.DK | IVb | 4 | 1 | 1 |
| 6 | GX6A16.0026.DK | GX6A12.0018.DK | | 4 | 1 | 7 |
| 6 | GX6A16.0027.DK | GX6A12.0020.DK | | 4 | 1 | 14 |
| 6 | GX6A16.0026.DK | GX6A12.0232.DK | | 4 | 1 | 5 |
| 6 | GX6A16.0026.DK | GX6A12.0020.DK | | 4 | 1 | 7 |
| 6 | GX6A16.0027.DK | GX6A12.0017.DK | | 4 | 2 | 6 |
| 6 | GX6A16.0027.DK | GX6A12.0018.DK | IVb | 4 | 2 | 8 |
| 330 | GX6A16.0091.DK | GX6A12.0112.DK | IIb | 1 | 1 | 1 |
| 224 | GX6A16.0181.DK | GX6A12.0218.DK | | 1 | 1 | 3 |
| | | | | Total | 92 | 387 |

**Technical Appendix Figure 2.** Dendogram based on MLST of the 53 PFGE profiles (pulsotypes) represented by the 92 isolates selected for MLST. ST, sequence type; Mol Sero, PCR-serogroup; Sero, serotype; No. MLST, number of isolates from the respective pulsotypes that were MLST-typed; Total no. pulsotype, the total number of isolates with the respective pulsotype in the study.
